# Investigation of solid-state photochemical nitro–nitrito linkage isomerization: crystal structures of *trans*-bis­(ethyl­enedi­amine)(iso­thio­cyanato)­nitritocobalt(III) salts: thio­cyanate, chloride monohydrate, and perchlorate–thio­cyanate­(0.75/0.25)

**DOI:** 10.1107/S2056989018013634

**Published:** 2018-09-28

**Authors:** Shigeru Ohba, Masanobu Tsuchimoto, Saeko Kurachi

**Affiliations:** a Research and Education Center for Natural Sciences, Keio University, Hiyoshi 4-1-1, Kohoku-ku, Yokohama 223-8521, Japan; b Department of Chemistry, Chiba Institute of Technology, Shibazono 2-1-1, Narashino, Chiba 275-0023, Japan; c Department of Chemistry, Faculty of Science and Technology, Keio University, Hiyoshi 3-14-1, Kohoku-ku, Yokohama 223-8522, Japan

**Keywords:** crystal structure, complex ion, nitro–nitrito photo-isomerization, reaction cavity

## Abstract

The reaction cavities of the nitro groups in the crystals of the title compounds, *trans*-[Co(NO_2_)(NCS)(C_2_H_8_N_2_)_2_]·*X*, *X* = SCN^−^ (I), Cl^−^·H_2_O (II), and (ClO_4_^−^)_0.75_(SCN^−^)_0.25_ (III), have been investigated, revealing that the geometry of the inter­molecular N—H⋯O hydrogen bonds in (I) is unsuitable for nitro–nitrito photo-isomerization. The common main building block of these crystal structures is a centrosymmetric pair of complex cations connected by pairwise N—H⋯O(nitro) hydrogen bonds forming an *R*_2_^2^(4) ring, which is a narrow diamond shape in (I) but is approximately square in (II) and (III). The structure of (I) was reported earlier [Börtin (1976 ▸). *Acta Chem. Scand. A*, **30**, 503–506] but is described here with an improved disorder model for the thio­cyanate anions and to higher precision.

## Abstract 2

The crystal structures of the title compounds have been studied to confirm that the solid-state photochemical nitro–nitrito linkage isomerization is restricted by the reaction cavity of the nitrite ion in the thio­cyanate salt.

## Chemical context   

The nitrite ion is one of the well-known ligands that show linkage isomerism even in the solid state (Hatcher & Raithby, 2013 ▸). Adell (1971 ▸) prepared *trans*-[Co(en)_2_(NO_2_)(NCS)]*X* (en = ethyl­enedi­amine, *X* = a counter-anion and a solvent mol­ecule if incorporated into the crystal structure) to show that irradiation by sunlight or visible light (λ > 430 nm) alters the color of the crystals from orange to red for perchlorate and nitrate salts, indicating nitro–nitrito photochemical isomerization, but not for thio­cyanate. These facts suggest that the photo-isomerization is inter­rupted by some steric condition in (I) where *X* = SCN^−^. Börtin (1976 ▸) determined the crystal structure of (I), but failed to find the steric obstacles to the reaction, and the puzzle has been left unsolved. Kubota & Ohba (1992 ▸) investigated the solid-state nitro–nitrito photochemical reaction of [Co(NH_3_)_5_NO_2_]Cl_2_ to show that the shape of the reaction cavity in the nitro plane is of crucial importance. It is noted that not only the steric condition around the nitro group, but also the electronic effects of the co-existing ligands are important for the longer lifetime of the much less stable nitrito form (Miyoshi *et al.*, 1983 ▸), the thio­cyanate ligand at the *trans* position being favorable. When the powders were irradiated by a 150 W Xe lamp without filtering, the color changed immediately from yellow to orange for (II) and (III) but not for (I), in agreement with the observations of Adell (1971 ▸). In the present study, the structures of the three title crystals were investigated to reveal the steric conditions that make (I) photo-inactive.
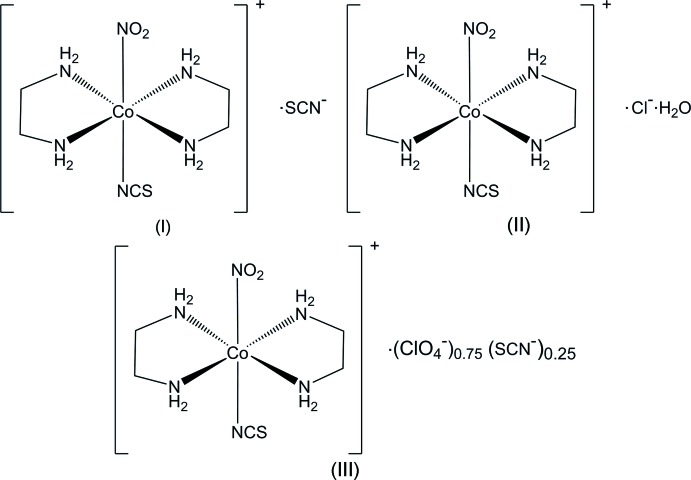


## Structural commentary   

**Figure 1:**
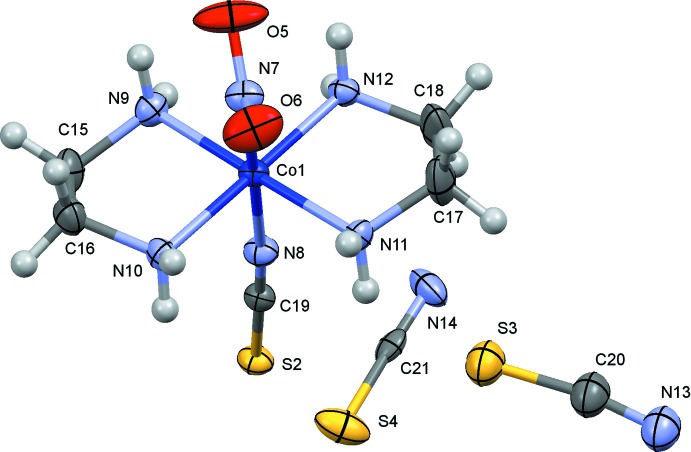
The mol­ecular structure of (I), showing displacement ellipsoids at the 30% probability level. Only one of two possible orientations of the disordered thio­cyanate (N13/C20/S3 and N14/C21/S4) ions is indicated for clarity.

The crystal structure of (I) has been redetermined in the present study with a more sophisticated treatment of the disorder of thio­cyanate ions [*R*(*F* ^2^) = 0.048 for 2845 observed reflections] than that reported by Börtin (1976 ▸) [*R*(*F*) = 0.077 for 1970 reflections], and the s.u.’s of the bond lengths were reduced to less than half of the previous values. The mol­ecular structures of (I)–(III) are shown in Figs. 1 ▸–3 ▸ ▸, respectively. The coordination geometry around the Co atoms is octa­hedral, and the Co—N(nitro) bond lengths are similar to one another, 1.905 (3) Å in (I), 1.912 (2) Å in (II) and 1.915 (4) and 1.916 (4) Å in (III). The conformations of the ethyl­ene­diammine ligands are *gauche* in (I) and (III), and envelope in (II). The short C17—C18 distance of 1.417 (8) Å in (I) may be an artifact of unresolved disorder over two orientations by the puckering of the chelate ring as mentioned by Börtin (1976 ▸). The combination of the two ethyl­enedi­amine chelate rings in each complex is δ and λ, and the Co(en)_2_ moiety possesses approximate mirror symmetry. In (I), there are two independent thio­cyanate counter-ions, which are disordered around twofold axes and are therefore half occupied. In (II), there is a chloride counter-ion and an ordered water mol­ecule of crystallization. In (III), one of the two perchlorate ions (Cl4/O16–O19) lies on a center of symmetry, showing orientational disorder. Furthermore, an unexpected thio­cyanate ion (S7/C43/N32) exists on a center of symmetry, possessing two possible orientations. The asymmetric unit of (III) comprises two complex cations, one and half perchlorate ions, and half a thio­cyanate ion.

## Supra­molecular features   

**Figure 4:**
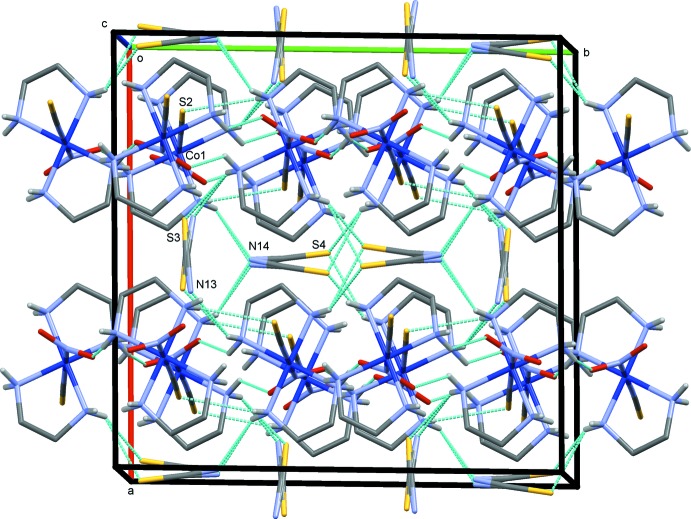
The crystal structure of (I), projected along *c*. N—H⋯O/N/S hydrogen bonds are shown as blue dashed lines. Both possible orientations of the disordered thio­cyanate ions are indicated.

**Figure 7:**
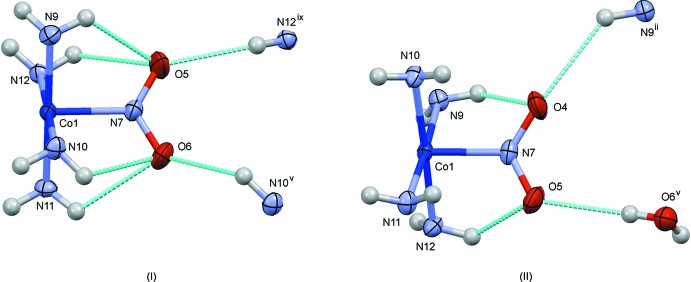
Comparison of the steric circumstances of the nitro group in (I) and (II). Dashed lines in blue indicate O(nitro)⋯H short contacts shorter than 2.5 Å. Only part of the di­amine ligands are shown for clarity. Symmetry codes for (I): (v) −*x* + 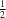, −*y* + 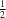, −*z*; (ix) *x*, −*y*, *z* − 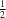. For (II): (ii) −*x* + 1, −*y* + 1, −*z* + 1; (v) *x* − 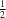, −*y* + 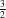, *z* + 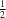.

**Figure 8:**
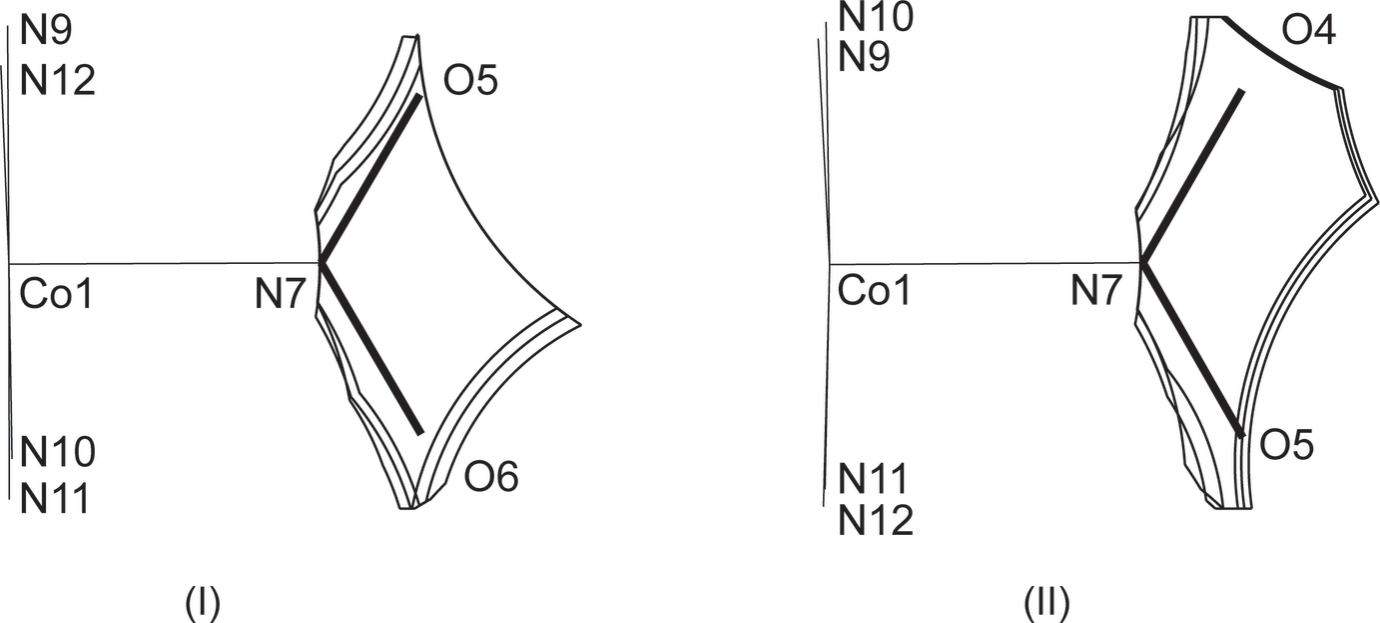
Comparison of the slices of the cavity around the nitro group within 0.1 Å from the plane in (I) and (II).

**Figure 9:**
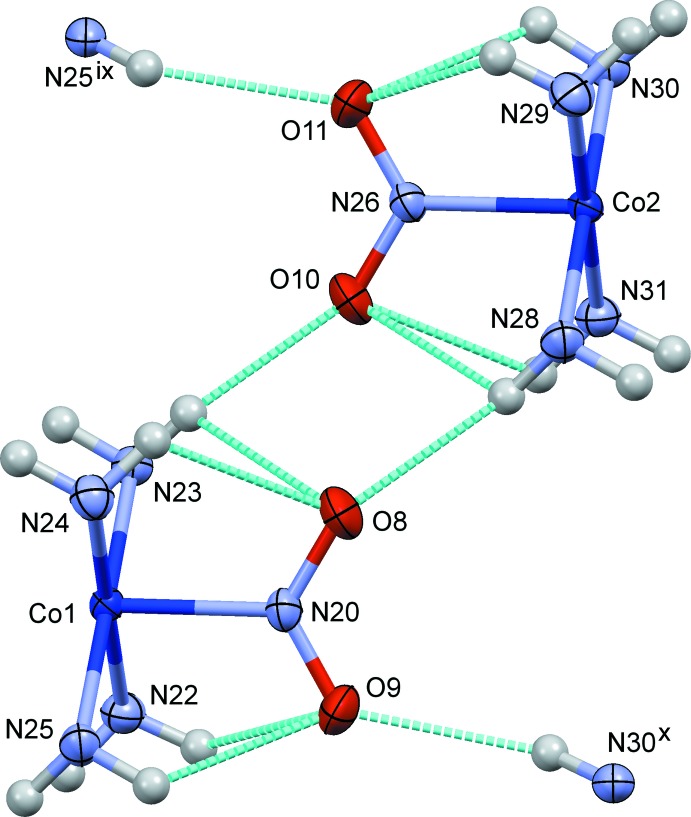
The steric circumstances of the nitro groups in (III). Dashed lines in blue show the O(nitro)⋯H short contacts shorter than 2.5 Å. Only parts of the di­amine ligands are shown for clarity. Symmetry codes: (ix) −*x* + 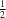, *y* + 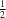, −*z* + 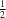; (x) −*x* + 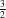, *y* − 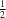, −*z* + 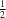.

**Figure 10:**
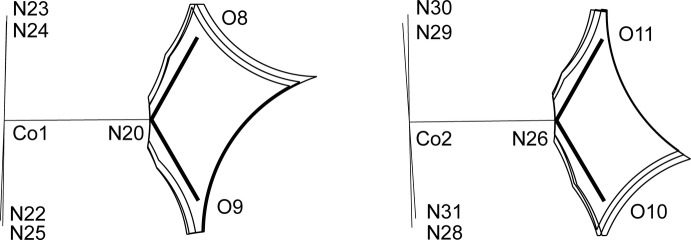
The slices of the cavity in (III) around the nitro groups within 0.1 Å from the planes.

**Figure 11:**
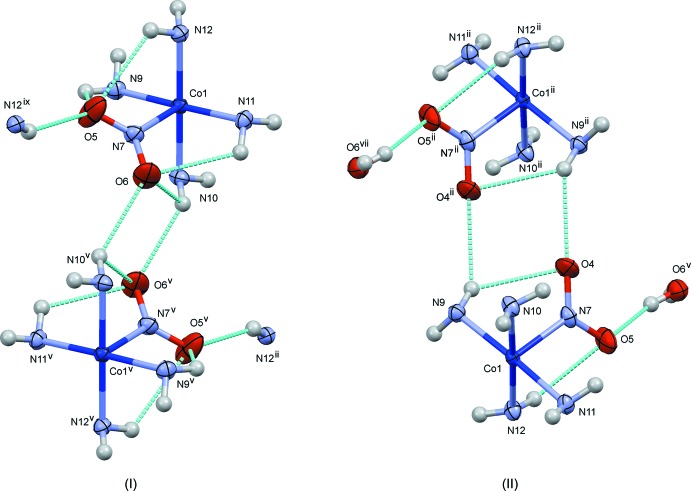
Comparison of the short contact pair of the nitro group in (I) and (II). Dashed lines in blue show the O(nitro)⋯H short contacts shorter than 2.5 Å. Only parts of the di­amine ligands are shown for clarity. Symmetry codes for (I): (ii) −*x* + 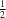, *y* − 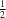, −*z* + 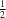; (v) −*x* + 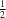, −*y* + 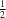, −*z*; (ix) *x*, −*y*, *z* − 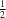. For (II): (ii) −*x* + 1, −*y* + 1, −*z* + 1, (v) *x* − 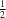, −*y* + 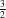, *z* + 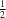; (vii) −*x* + 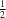, *y* + 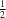, −*z* + 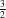.

The crystal structures of (I)–(III) are shown in Figs. 4 ▸–6 ▸ ▸, respectively. The complex cations and the counter-anions are connected *via* numerous hydrogen bonds (Tables 1 ▸–3 ▸ ▸), forming three-dimensional networks. The circumstances of the nitro groups in (I) and (II) are compared in Fig. 7 ▸, where the surrounding hydrogen-bond donors are projected on the nitro plane. The nitro O atoms act as acceptors of intra- and inter­molecular N/O—H⋯O hydrogen bonds. It is expected that the nitro–nitrito photo-isomerization occurs *via* an *N*,*O*-bidentate transition state (Johnson & Pashman, 1975 ▸) by rotating the nitrite ion in its original plane because of the feasible charge density due to the lone pairs of the nitrite N and O atoms (Okuda *et al.*, 1990 ▸). It seems that the *N*,*O*-bidentate mode is prevented by the inter­molecular N—H⋯O hydrogen bonds in (I), but it may be allowed in (II) because of the vacant space behind the nitro O4 atom. This can be seen from the slices of the cavity around the NO_2_^−^ group (Fig. 8 ▸), which is defined as the concave space limited by the envelope surfaces of spheres placed at the positions of neighboring atoms, each sphere having a radius 1.0 Å greater (as selected by Kubota & Ohba, 1992 ▸) than the corresponding van der Waals radius (Bondi, 1964 ▸) except for the Co, its radius being assumed to be 1.90 Å, which is a little shorter than the Co—N(nitro) distance. Asymmetric inter­molecular hydrogen-bond contacts are also observed in (III) (Fig. 9 ▸), and the reaction cavities show the vacancy at one of the two O atoms, O8 and O10 (Fig. 10 ▸). The 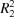
(4) ring formed by the pair of nitro groups is observed not only in (III) but also in (I) and (II) (Fig. 11 ▸). These four-membered rings are essentially planar with the O⋯H distances ranging from 2.33 to 2.49 Å. However, there are apparent differences in the geometry. That in (I) is a narrow rhomb with the inter­ior angles at O6 and H10*B* being 33.3 and 146.7°, respectively, and inclined to the nitro plane by 79.2 (3)°. The corresponding angles at O4 and H9*A* in (II) are 98.7 and 81.3°, and the dihedral angle with the nitro plane is 45.5 (2)°. The shape of the ring in (III) is also nearly square with inter­ior angles of 87.3–92.4°, and the dihedral angles with the nitro planes are 53.6 (2) and 53.8 (2)°.

## Database survey   

Grenthe & Nordin (1979 ▸) reported the structures of *trans*-{Co(en)_2_(NO_2_)(NCS)]·*X* (*X* = ClO_4_^−^ and I^−^) obtained after solid-state thermal isomerization of the nitrito complexes (monoclinic *P*2_1_, *Z* = 2). The lattice constants did not correspond to the crystals grown from aqueous solutions of the nitro complexes. Except for Börtin (1976 ▸) (*X* = SCN^−^) there is no other entry of the title nitro­cobalt complex in the Cambridge Structural Database (CSD Version 5.39; Groom *et al.*, 2016 ▸).

## Synthesis and crystallization   

The title thio­cyanate salt (I) was prepared by a literature method (Adell, 1971 ▸; Nakahara & Shibata, 1977 ▸) from cobalt(II) nitrate hexa­hydrate *via trans*-[Co(en)_2_(NO_2_)_2_]NO_3_ and then *trans*-[Co(en)_2_Cl(NO_2_)]NO_3_. The crystals of (I) were grown from a hot aqueous solution. Crystals of (I) were pulverized and dissolved in conc. HCl over a moderate heat, and impurities were removed by filtration. To the filtrate, some amount of ethanol was added. The solution was concentrated to precipitate the chloride (II), which was recrystallized with a small amount of water as solvent. To the saturated aqueous solution of (II), NaClO_4_ powder was added to precipitate the perchlorate (III). Crystals of (III) were grown from an aqueous solution. The possibility of contamination of (III) by chloride ions was eliminated because no precipitation of AgCl occurred when AgNO_3_ was added to an aqueous solution.

## Refinement   

Crystal data, data collection and structure refinement details are summarized in Table 4 ▸. The H atoms bound to C and N were positioned geometrically. They were refined as riding, with N—H = 0.89 Å, C—H = 0.97 Å, and *U*_iso_(H) = 1.2*U*_eq_(C/N).

In (I), the non-coordinating thio­cyanate ions S3/C20/N13 and S4/C21/N14 lie around the twofold axis with the mol­ecular axes perpendicular and slightly inclined, respectively, showing orientational disorder. Their geometries were restrained with EADP or SIMU commands. Three reflections showing very poor agreement with *I*_obs_ much smaller than *I*_calc_ were omitted from the final refinement.

In (II), the H atoms of the water mol­ecule were located from difference-density maps, and their coordinates were refined with the geometry restrained, and with *U*_iso_(H) = 1.5*U*_eq_(O). Eight reflections showing poor agreement were omitted from the final refinement, since their *I*_obs_ were much smaller than *I*_calc_.

In (III), atom Cl4 of one of the two independent perchlor­ate ions lies on a center of symmetry, showing orientational disorder. Another independent and indistinct anion lies over the center of symmetry, but is not a perchlorate ion since the electron-density peaks of the O atoms are missing. It is not a chloride ion either, judging from the lack of precipitation of AgCl with silver nitrate. The most probable and suitable assumption is that the thio­cyanate ion has two possible orientations as seen in (I), and the expected composition is supported by the measured density of the crystals, 1.76 (2) Mg m^−3^, which agrees well with the calculated value, 1.759 Mg m^−3^. The geometry of the disordered thio­cyanate ion was restrained with an EADP instruction for the terminal S7/N32 atoms and DELU and ISOR instructions for the central C43 atom to avoid the abnormally large residual peak near the C43 atom. One reflection with *I*_obs_ much smaller than *I*_calc_ was omitted from the final refinement.

## Supplementary Material

Crystal structure: contains datablock(s) I, II, III, general. DOI: 10.1107/S2056989018013634/hb7774sup1.cif

Structure factors: contains datablock(s) I. DOI: 10.1107/S2056989018013634/hb7774Isup2.hkl

Supporting information file. DOI: 10.1107/S2056989018013634/hb7774Isup5.cdx

Structure factors: contains datablock(s) II. DOI: 10.1107/S2056989018013634/hb7774IIsup3.hkl

Supporting information file. DOI: 10.1107/S2056989018013634/hb7774IIsup6.cdx

Structure factors: contains datablock(s) III. DOI: 10.1107/S2056989018013634/hb7774IIIsup4.hkl

Supporting information file. DOI: 10.1107/S2056989018013634/hb7774IIIsup7.cdx

CCDC references: 1869545, 1869544, 1869543

Additional supporting information:  crystallographic information; 3D view; checkCIF report

## Figures and Tables

**Figure 2:**
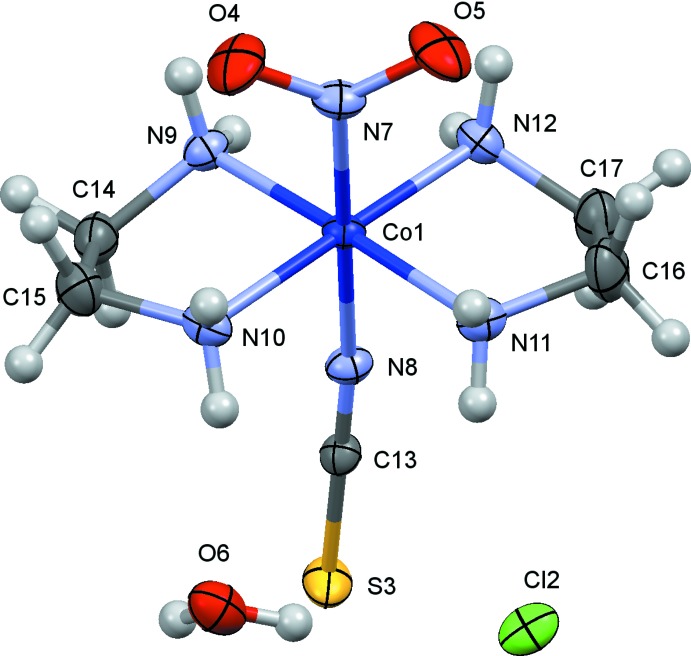
The mol­ecular structure of (II), showing displacement ellipsoids at the 30% probability level.

**Figure 3:**
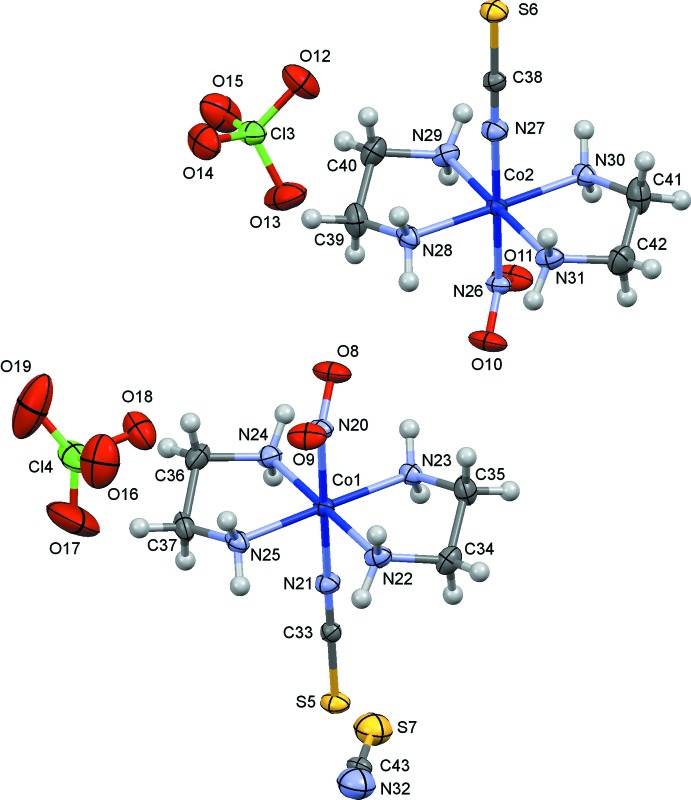
The mol­ecular structure of (III), showing displacement ellipsoids at the 30% probability level. Only one of two possible orientations of the disordered thio­cyanate (S7/C43/N32) and perchlorate (Cl4/O16–O19) ions is indicated for clarity.

**Figure 5:**
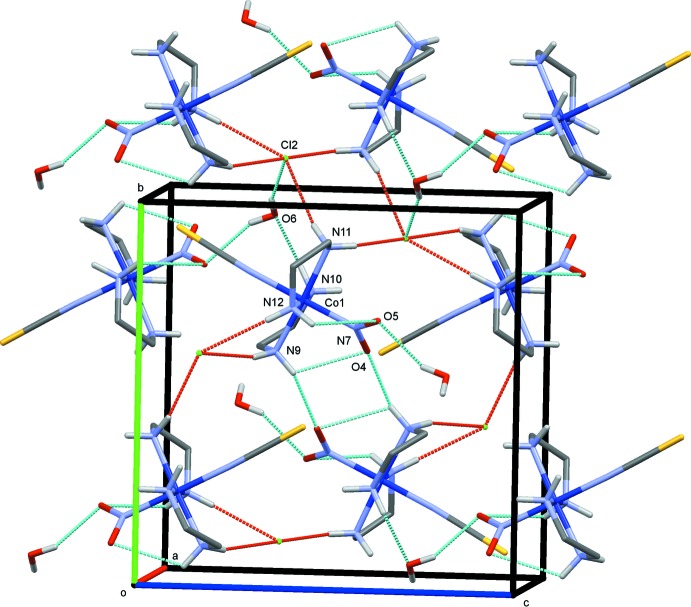
The crystal structure of (II), projected along *a*. Hydrogen bonds are shown as dashed lines in blue for O—H⋯O/Cl and N—H⋯O, and in red for N—H⋯Cl.

**Figure 6:**
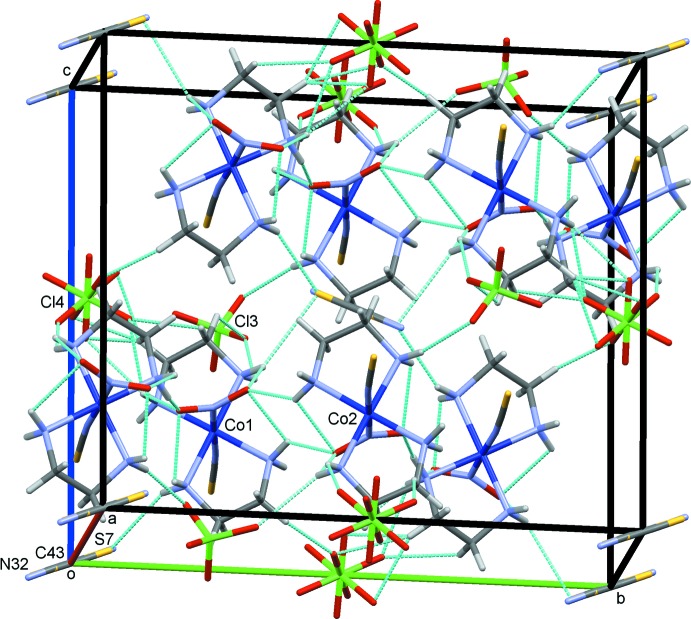
The crystal structure of (III), projected along *a*. N—H⋯O/N and C—H⋯O hydrogen bonds are shown as blue dashed lines. Both possible orientations of the disordered thio­cyanate (S7/C43/N32) and perchlorate (Cl4/O16–O19) ions are indicated.

**Table 1:** Hydrogen-bond geometry (Å, °) for (I)

*D*—H⋯*A*	*D*—H	H⋯*A*	*D*⋯*A*	*D*—H⋯*A*
N9—H9*A*⋯O5	0.89	2.31	2.861 (5)	120
N9—H9*B*⋯S4^i^	0.89	2.75	3.443 (4)	136
N9—H9*B*⋯S4^ii^	0.89	2.58	3.404 (4)	154
N10—H10*A*⋯S3^iii^	0.89	2.73	3.480 (7)	143
N10—H10*A*⋯N13^iv^	0.89	2.63	3.41 (2)	147
N10—H10*B*⋯O6	0.89	2.49	2.950 (5)	113
N10—H10*B*⋯O6^v^	0.89	2.33	3.013 (4)	133
N11—H11*A*⋯O6	0.89	2.40	2.869 (5)	113
N11—H11*A*⋯N14	0.89	2.53	3.28 (3)	142
N11—H11*A*⋯N14^vi^	0.89	2.36	3.12 (2)	143
N11—H11*B*⋯S2^iii^	0.89	2.77	3.360 (3)	125
N12—H12*A*⋯O5^vii^	0.89	2.23	3.011 (4)	146
N12—H12*B*⋯O5	0.89	2.47	2.984 (5)	117
C15—H15*A*⋯S2^viii^	0.97	2.83	3.731 (4)	155
C18—H18*B*⋯S3^ix^	0.97	2.87	3.627 (8)	136

Symmetry codes: (i) 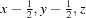; (ii) 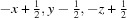; (iii) 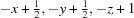; (iv) 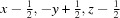; (v) 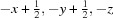; (vi) 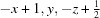; (vii) 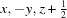; (viii) 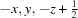; (ix) 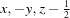.

**Table 2:** Hydrogen-bond geometry (Å, °) for (II)

*D*—H⋯*A*	*D*—H	H⋯*A*	*D*⋯*A*	*D*—H⋯*A*
O6—H6*A*⋯O5^i^	0.82 (2)	2.19 (2)	2.958 (3)	156 (4)
O6—H6*B*⋯Cl2	0.83 (2)	2.45 (2)	3.253 (3)	162 (4)
N9—H9*A*⋯O4	0.89	2.49	2.960 (3)	114
N9—H9*A*⋯O4^ii^	0.89	2.48	3.191 (3)	138
N9—H9*B*⋯Cl2^iii^	0.89	2.43	3.297 (2)	165
N10—H10*A*⋯O6	0.89	2.08	2.960 (3)	171
N10—H10*B*⋯Cl2^iv^	0.89	2.75	3.461 (2)	138
N11—H11*A*⋯Cl2^iv^	0.89	2.44	3.260 (2)	153
N11—H11*B*⋯Cl2	0.89	2.42	3.285 (2)	164
N12—H12*A*⋯Cl2^iii^	0.89	2.47	3.335 (2)	164
N12—H12*B*⋯S3^v^	0.89	2.75	3.585 (2)	157
N12—H12*B*⋯O5	0.89	2.41	2.887 (3)	114
C15—H15*B*⋯S3^vi^	0.97	2.77	3.546 (3)	138

Symmetry codes: (i) 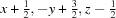; (ii) 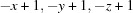; (iii) 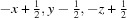; (iv) 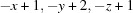; (v) 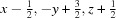; (vi) 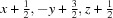.

**Table 3:** Hydrogen-bond geometry (Å, °) for (III)

*D*—H⋯*A*	*D*—H	H⋯*A*	*D*⋯*A*	*D*—H⋯*A*
N22—H22*A*⋯O9	0.89	2.39	2.925 (5)	119
N22—H22*B*⋯S7	0.89	2.64	3.456 (12)	153
N23—H23*A*⋯S6^i^	0.89	2.82	3.429 (4)	127
N23—H23*B*⋯O8	0.89	2.41	2.884 (5)	113
N23—H23*B*⋯O10	0.89	2.34	3.021 (5)	133
N24—H24*A*⋯S7^ii^	0.89	2.79	3.475 (18)	135
N24—H24*A*⋯O8	0.89	2.41	2.875 (6)	113
N24—H24*B*⋯S6^i^	0.89	2.84	3.437 (4)	126
N24—H24*B*⋯O12^i^	0.89	2.53	3.192 (6)	132
N25—H25*A*⋯O11^iii^	0.89	2.28	3.059 (5)	147
N25—H25*B*⋯O9	0.89	2.45	2.973 (5)	118
N25—H25*B*⋯O18	0.89	2.56	3.237 (12)	133
N25—H25*B*⋯O19^iv^	0.89	2.44	3.089 (15)	130
N28—H28*A*⋯O8	0.89	2.36	3.057 (5)	135
N28—H28*A*⋯O10	0.89	2.45	2.915 (6)	113
N28—H28*B*⋯O13	0.89	2.48	3.107 (6)	128
N29—H29*A*⋯O15^v^	0.89	2.39	3.266 (7)	170
N29—H29*B*⋯O11	0.89	2.38	2.919 (6)	119
N30—H30*A*⋯O11	0.89	2.44	2.967 (6)	118
N30—H30*B*⋯O9^vi^	0.89	2.30	3.067 (5)	144
N31—H31*A*⋯S5^vii^	0.89	2.74	3.343 (4)	126
N31—H31*A*⋯O16^vi^	0.89	2.57	3.294 (17)	139
N31—H31*B*⋯O10	0.89	2.40	2.856 (6)	112
N31—H31*B*⋯O14^viii^	0.89	2.45	3.172 (6)	139
C35—H35*B*⋯O17^ix^	0.97	2.24	3.09 (2)	145
C36—H36*A*⋯O12^i^	0.97	2.56	3.119 (8)	117
C36—H36*B*⋯O18	0.97	2.53	3.249 (11)	131
C37—H37*A*⋯O16^iv^	0.97	2.48	3.290 (15)	141
C37—H37*A*⋯O19^iv^	0.97	2.55	3.197 (17)	124
C39—H39*A*⋯O13	0.97	2.44	3.133 (8)	128
C41—H41*A*⋯O13^vi^	0.97	2.38	3.202 (8)	143
C41—H41*B*⋯O18^vi^	0.97	2.37	3.272 (12)	155

Symmetry codes: (i) 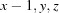; (ii) 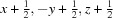; (iii) 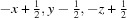; (iv) 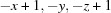; (v) 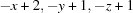; (vi) 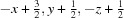; (vii) 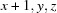; (viii) 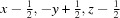; (ix) 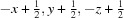.

**Table 4:** Experimental details

	(I)	(II)	(III)
Crystal data			
Chemical formula	[Co(NCS)(NO_2_)(C_2_H_8_N_2_)_2_](CNS)	[Co(NCS)(NO_2_)(C_2_H_8_N_2_)_2_]Cl·H_2_O	[Co(NCS)(NO_2_)(C_2_H_8_N_2_)_2_](ClO_4_)_0.75_(CNS)_0.25_
*M*_r_	341.31	336.69	372.33
Crystal system, space group	Monoclinic, *C*2/*c*	Monoclinic, *P*2_1_/*n*	Monoclinic, *P*2_1_/*n*
Temperature (K)	299	301	301
*a*, *b*, *c* (Å)	16.3222 (7), 16.0137 (6), 11.1284 (4)	8.9059 (4), 12.3302 (5), 12.2915 (5)	11.3141 (6), 16.2969 (7), 16.1298 (7)
β (°)	110.2599 (13)	92.295 (2)	109.023 (2)
*V* (Å^3^)	2728.77 (19)	1348.67 (10)	2811.7 (2)
*Z*	8	4	8
Radiation type	Mo *K*α	Mo *K*α	Mo *K*α
μ (mm^−1^)	1.57	1.63	1.58
Crystal size (mm)	0.25 × 0.25 × 0.20	0.25 × 0.20 × 0.20	0.35 × 0.30 × 0.27

Data collection			
Diffractometer	Bruker D8 VENTURE	Bruker D8 VENTURE	Bruker D8 VENTURE
Absorption correction	Integration (*SADABS*; Bruker, 2016 ▸)	Integration (*SADABS*; Bruker, 2016 ▸)	Integration (*SADABS*; Bruker, 2016 ▸)
*T*_min_, *T*_max_	0.667, 0.743	0.697, 0.762	0.544, 0.774
No. of measured, independent and observed [*I* > 2σ(*I*)] reflections	14627, 3166, 2845	14312, 3153, 2830	30435, 6595, 5411
*R*_int_	0.023	0.024	0.034
(sin θ/λ)_max_ (Å^−1^)	0.659	0.659	0.659

Refinement			
*R*[*F* ^2^ > 2σ(*F* ^2^)], *wR*(*F* ^2^), *S*	0.048, 0.173, 1.06	0.032, 0.131, 0.98	0.063, 0.221, 1.10
No. of reflections	3166	3153	6595
No. of parameters	179	161	379
No. of restraints	18	3	13
H-atom treatment	H-atom parameters constrained	H atoms treated by a mixture of independent and constrained refinement	H-atom parameters constrained
Δρ_max_, Δρ_min_ (e Å^−3^)	0.99, −0.92	0.74, −0.54	1.40, −1.15

Computer programs: *APEX3* and *SAINT* (Bruker, 2016 ▸), *SHELXT* (Sheldrick, 2015*a* ▸), *SHELXL2014* (Sheldrick, 2015*b* ▸), *Mercury* (Macrae *et al.*, 2008 ▸), *CAVITY* (Ohashi *et al.*, 1981 ▸) and *publCIF* (Westrip, 2010 ▸).

## supplementary crystallographic information

### trans-Bis(ethylenediamine)(isothiocyanato)nitritocobalt(III) thiocyanate (I) Crystal data

**Table:**

[Co(CNS)(NO_2_)(C_2_H_8_N_2_)_2_](CNS)	*F*(000) = 1408
*M**_r_* = 341.31	*D*_x_ = 1.662 Mg m^−^^3^*D*_m_ = 1.65 (2) Mg m^−^^3^*D*_m_ measured by flotation
Monoclinic, *C*2/*c*	Mo *K*α radiation, λ = 0.71073 Å
*a* = 16.3222 (7) Å	Cell parameters from 9404 reflections
*b* = 16.0137 (6) Å	θ = 2.5–27.9°
*c* = 11.1284 (4) Å	µ = 1.57 mm^−^^1^
β = 110.2599 (13)°	*T* = 299 K
*V* = 2728.77 (19) Å^3^	Prism, orange-red
*Z* = 8	0.25 × 0.25 × 0.20 mm

### trans-Bis(ethylenediamine)(isothiocyanato)nitritocobalt(III) thiocyanate (I) Data collection

**Table:**

Bruker D8 VENTURE diffractometer	2845 reflections with *I* > 2σ(*I*)
φ and ω scans	*R*_int_ = 0.023
Absorption correction: integration (SADABS; Bruker, 2016)	θ_max_ = 27.9°, θ_min_ = 2.3°
*T*_min_ = 0.667, *T*_max_ = 0.743	*h* = −21→17
14627 measured reflections	*k* = −19→21
3166 independent reflections	*l* = −14→14

### trans-Bis(ethylenediamine)(isothiocyanato)nitritocobalt(III) thiocyanate (I) Refinement

**Table:**

Refinement on *F*^2^	Hydrogen site location: inferred from neighbouring sites
Least-squares matrix: full	H-atom parameters constrained
*R*[*F*^2^ > 2σ(*F*^2^)] = 0.048	*w* = 1/[σ^2^(*F*_o_^2^) + (0.1131*P*)^2^ + 7.7542*P*] where *P* = (*F*_o_^2^ + 2*F*_c_^2^)/3
*wR*(*F*^2^) = 0.173	(Δ/σ)_max_ = 0.004
*S* = 1.06	Δρ_max_ = 0.99 e Å^−^^3^
3166 reflections	Δρ_min_ = −0.92 e Å^−^^3^
179 parameters	Extinction correction: SHELXL2014 (Sheldrick, 2015b), Fc^*^=kFc[1+0.001xFc^2^λ^3^/sin(2θ)]^-1/4^
18 restraints	Extinction coefficient: 0.0080 (10)
Primary atom site location: structure-invariant direct methods	

### trans-Bis(ethylenediamine)(isothiocyanato)nitritocobalt(III) thiocyanate (I) Special details

**Table:**

Geometry. All esds (except the esd in the dihedral angle between two l.s. planes) are estimated using the full covariance matrix. The cell esds are taken into account individually in the estimation of esds in distances, angles and torsion angles; correlations between esds in cell parameters are only used when they are defined by crystal symmetry. An approximate (isotropic) treatment of cell esds is used for estimating esds involving l.s. planes.

### trans-Bis(ethylenediamine)(isothiocyanato)nitritocobalt(III) thiocyanate (I) Fractional atomic coordinates and isotropic or equivalent isotropic displacement parameters (Å^2^)

**Table:**

	*x*	*y*	*z*	*U*_iso_*/*U*_eq_	Occ. (<1)
Co1	0.24789 (3)	0.11825 (3)	0.23306 (4)	0.0283 (2)	
S2	0.16608 (6)	0.13940 (6)	0.59617 (8)	0.0395 (3)	
S3	0.4182 (2)	0.1460 (3)	0.6750 (5)	0.0699 (8)	0.5
S4	0.4754 (2)	0.45097 (14)	0.2044 (3)	0.0701 (8)	0.5
O5	0.2530 (4)	0.0509 (3)	0.0120 (4)	0.1008 (17)	
O6	0.3109 (3)	0.1679 (3)	0.0477 (4)	0.0816 (12)	
N7	0.2733 (2)	0.1111 (2)	0.0787 (3)	0.0404 (7)	
N8	0.2210 (2)	0.12697 (17)	0.3870 (3)	0.0388 (7)	
N9	0.13307 (19)	0.0704 (2)	0.1463 (3)	0.0403 (6)	
H9A	0.1308	0.0480	0.0720	0.048*	
H9B	0.1223	0.0304	0.1943	0.048*	
N10	0.19177 (19)	0.22653 (18)	0.1777 (3)	0.0379 (6)	
H10A	0.1885	0.2544	0.2451	0.045*	
H10B	0.2235	0.2566	0.1426	0.045*	
N11	0.36443 (17)	0.16416 (19)	0.3219 (3)	0.0379 (6)	
H11A	0.3784	0.2000	0.2710	0.045*	
H11B	0.3649	0.1915	0.3918	0.045*	
N12	0.30175 (19)	0.00934 (17)	0.2894 (3)	0.0357 (6)	
H12A	0.2704	−0.0189	0.3271	0.043*	
H12B	0.3027	−0.0199	0.2219	0.043*	
N13	0.5878 (6)	0.1646 (14)	0.8455 (16)	0.0699 (8)	0.5
N14	0.5058 (18)	0.2805 (4)	0.248 (3)	0.061 (3)	0.5
C15	0.0661 (3)	0.1389 (3)	0.1234 (5)	0.0552 (11)	
H15A	0.0121	0.1227	0.0568	0.066*	
H15B	0.0538	0.1502	0.2012	0.066*	
C16	0.1033 (3)	0.2141 (3)	0.0836 (4)	0.0537 (10)	
H16A	0.1060	0.2062	−0.0013	0.064*	
H16B	0.0672	0.2625	0.0819	0.064*	
C17	0.4281 (3)	0.0962 (3)	0.3576 (6)	0.0694 (15)	
H17A	0.4519	0.0880	0.2898	0.083*	
H17B	0.4759	0.1117	0.4346	0.083*	
C18	0.3910 (3)	0.0202 (3)	0.3795 (7)	0.087 (2)	
H18A	0.3905	0.0197	0.4664	0.105*	
H18B	0.4268	−0.0261	0.3706	0.105*	
C19	0.1993 (2)	0.13285 (19)	0.4742 (3)	0.0323 (7)	
C20	0.5197 (4)	0.1522 (7)	0.7695 (10)	0.0699 (8)	0.5
C21	0.4903 (12)	0.3510 (3)	0.2317 (12)	0.039 (3)	0.5

### trans-Bis(ethylenediamine)(isothiocyanato)nitritocobalt(III) thiocyanate (I) Atomic displacement parameters (Å^2^)

**Table:**

	*U*^11^	*U*^22^	*U*^33^	*U*^12^	*U*^13^	*U*^23^
Co1	0.0326 (3)	0.0277 (3)	0.0275 (3)	−0.00172 (14)	0.0139 (2)	0.00089 (13)
S2	0.0484 (5)	0.0413 (5)	0.0350 (5)	0.0026 (3)	0.0225 (4)	−0.0027 (3)
S3	0.0537 (11)	0.090 (3)	0.063 (2)	0.0010 (13)	0.0167 (11)	−0.0041 (15)
S4	0.098 (2)	0.0466 (11)	0.095 (2)	0.0011 (11)	0.0704 (18)	−0.0035 (10)
O5	0.182 (5)	0.077 (3)	0.082 (3)	−0.052 (3)	0.096 (3)	−0.041 (2)
O6	0.113 (3)	0.087 (3)	0.064 (2)	−0.041 (2)	0.055 (2)	0.0007 (19)
N7	0.0512 (18)	0.0401 (15)	0.0365 (16)	−0.0047 (13)	0.0234 (13)	0.0018 (11)
N8	0.0469 (18)	0.0393 (16)	0.0328 (14)	0.0005 (11)	0.0171 (13)	0.0005 (11)
N9	0.0382 (14)	0.0425 (16)	0.0421 (16)	−0.0056 (12)	0.0163 (12)	0.0004 (12)
N10	0.0434 (15)	0.0337 (14)	0.0372 (14)	0.0065 (11)	0.0147 (12)	0.0083 (11)
N11	0.0306 (13)	0.0399 (15)	0.0403 (15)	−0.0061 (11)	0.0086 (11)	−0.0033 (12)
N12	0.0421 (15)	0.0307 (13)	0.0342 (13)	0.0014 (11)	0.0129 (11)	−0.0001 (10)
N13	0.0537 (11)	0.090 (3)	0.063 (2)	0.0010 (13)	0.0167 (11)	−0.0041 (15)
N14	0.076 (8)	0.056 (4)	0.055 (3)	0.035 (8)	0.026 (5)	0.009 (9)
C15	0.0320 (19)	0.067 (3)	0.063 (3)	0.0023 (17)	0.0127 (18)	0.007 (2)
C16	0.049 (2)	0.047 (2)	0.058 (2)	0.0099 (17)	0.0088 (18)	0.0082 (18)
C17	0.0308 (19)	0.060 (3)	0.104 (4)	0.0011 (19)	0.006 (2)	0.015 (3)
C18	0.055 (3)	0.047 (3)	0.129 (5)	0.015 (2)	−0.008 (3)	0.007 (3)
C19	0.0352 (16)	0.0303 (15)	0.0329 (16)	0.0005 (11)	0.0136 (13)	−0.0005 (11)
C20	0.0537 (11)	0.090 (3)	0.063 (2)	0.0010 (13)	0.0167 (11)	−0.0041 (15)
C21	0.036 (9)	0.059 (4)	0.018 (8)	−0.011 (4)	0.005 (6)	−0.004 (3)

### trans-Bis(ethylenediamine)(isothiocyanato)nitritocobalt(III) thiocyanate (I) Geometric parameters (Å, º)

**Table:**

Co1—N7	1.905 (3)	N11—C17	1.462 (6)
Co1—N8	1.915 (3)	N11—H11A	0.8900
Co1—N9	1.944 (3)	N11—H11B	0.8900
Co1—N12	1.956 (3)	N12—C18	1.466 (6)
Co1—N11	1.958 (3)	N12—H12A	0.8900
Co1—N10	1.958 (3)	N12—H12B	0.8900
S2—C19	1.630 (3)	N13—C20	1.157 (4)
S3—C20	1.629 (3)	N14—C21	1.156 (4)
S4—C21	1.633 (3)	C15—C16	1.484 (6)
O5—N7	1.191 (5)	C15—H15A	0.9700
O6—N7	1.212 (4)	C15—H15B	0.9700
N8—C19	1.146 (5)	C16—H16A	0.9700
N9—C15	1.507 (5)	C16—H16B	0.9700
N9—H9A	0.8900	C17—C18	1.417 (8)
N9—H9B	0.8900	C17—H17A	0.9700
N10—C16	1.474 (5)	C17—H17B	0.9700
N10—H10A	0.8900	C18—H18A	0.9700
N10—H10B	0.8900	C18—H18B	0.9700
			
N7—Co1—N8	179.03 (13)	Co1—N11—H11B	109.8
N7—Co1—N9	90.11 (14)	H11A—N11—H11B	108.2
N8—Co1—N9	89.61 (14)	C18—N12—Co1	110.1 (3)
N7—Co1—N12	91.22 (13)	C18—N12—H12A	109.6
N8—Co1—N12	89.72 (12)	Co1—N12—H12A	109.6
N9—Co1—N12	93.23 (13)	C18—N12—H12B	109.6
N7—Co1—N11	90.05 (14)	Co1—N12—H12B	109.6
N8—Co1—N11	90.25 (14)	H12A—N12—H12B	108.2
N9—Co1—N11	178.80 (13)	C16—C15—N9	107.1 (3)
N12—Co1—N11	85.57 (12)	C16—C15—H15A	110.3
N7—Co1—N10	89.64 (13)	N9—C15—H15A	110.3
N8—Co1—N10	89.41 (12)	C16—C15—H15B	110.3
N9—Co1—N10	85.84 (13)	N9—C15—H15B	110.3
N12—Co1—N10	178.74 (12)	H15A—C15—H15B	108.6
N11—Co1—N10	95.35 (12)	N10—C16—C15	107.4 (3)
O5—N7—O6	119.3 (4)	N10—C16—H16A	110.2
O5—N7—Co1	120.6 (3)	C15—C16—H16A	110.2
O6—N7—Co1	120.1 (3)	N10—C16—H16B	110.2
C19—N8—Co1	175.5 (3)	C15—C16—H16B	110.2
C15—N9—Co1	108.3 (2)	H16A—C16—H16B	108.5
C15—N9—H9A	110.0	C18—C17—N11	112.3 (4)
Co1—N9—H9A	110.0	C18—C17—H17A	109.1
C15—N9—H9B	110.0	N11—C17—H17A	109.1
Co1—N9—H9B	110.0	C18—C17—H17B	109.1
H9A—N9—H9B	108.4	N11—C17—H17B	109.1
C16—N10—Co1	109.9 (2)	H17A—C17—H17B	107.9
C16—N10—H10A	109.7	C17—C18—N12	111.5 (4)
Co1—N10—H10A	109.7	C17—C18—H18A	109.3
C16—N10—H10B	109.7	N12—C18—H18A	109.3
Co1—N10—H10B	109.7	C17—C18—H18B	109.3
H10A—N10—H10B	108.2	N12—C18—H18B	109.3
C17—N11—Co1	109.4 (3)	H18A—C18—H18B	108.0
C17—N11—H11A	109.8	N8—C19—S2	178.4 (4)
Co1—N11—H11A	109.8	N13—C20—S3	170.7 (13)
C17—N11—H11B	109.8	N14—C21—S4	175 (2)
			
Co1—N9—C15—C16	40.7 (4)	N9—Co1—N10—C16	−11.2 (3)
Co1—N10—C16—C15	36.7 (4)	N12—Co1—N11—C17	10.8 (3)
N9—C15—C16—N10	−50.2 (5)	N11—Co1—N12—C18	8.7 (4)
Co1—N11—C17—C18	−29.4 (6)	O5—N7—Co1—N9	−41.7 (4)
N11—C17—C18—N12	37.6 (8)	O5—N7—Co1—N12	51.5 (5)
Co1—N12—C18—C17	−27.4 (7)	O6—N7—Co1—N10	52.8 (4)
N10—Co1—N9—C15	−16.5 (3)	O6—N7—Co1—N11	−42.6 (4)

### trans-Bis(ethylenediamine)(isothiocyanato)nitritocobalt(III) thiocyanate (I) Hydrogen-bond geometry (Å, º)

**Table:**

*D*—H···*A*	*D*—H	H···*A*	*D*···*A*	*D*—H···*A*
N9—H9*A*···O5	0.89	2.31	2.861 (5)	120
N9—H9*B*···S4^i^	0.89	2.75	3.443 (4)	136
N9—H9*B*···S4^ii^	0.89	2.58	3.404 (4)	154
N10—H10*A*···S3^iii^	0.89	2.73	3.480 (7)	143
N10—H10*A*···N13^iv^	0.89	2.63	3.41 (2)	147
N10—H10*B*···O6	0.89	2.49	2.950 (5)	113
N10—H10*B*···O6^v^	0.89	2.33	3.013 (4)	133
N11—H11*A*···O6	0.89	2.40	2.869 (5)	113
N11—H11*A*···N14	0.89	2.53	3.28 (3)	142
N11—H11*A*···N14^vi^	0.89	2.36	3.12 (2)	143
N11—H11*B*···S2^iii^	0.89	2.77	3.360 (3)	125
N12—H12*A*···O5^vii^	0.89	2.23	3.011 (4)	146
N12—H12*B*···O5	0.89	2.47	2.984 (5)	117
C15—H15*A*···S2^viii^	0.97	2.83	3.731 (4)	155
C18—H18*B*···S3^ix^	0.97	2.87	3.627 (8)	136

Symmetry codes: (i) *x*−1/2, *y*−1/2, *z*; (ii) −*x*+1/2, *y*−1/2, −*z*+1/2; (iii) −*x*+1/2, −*y*+1/2, −*z*+1; (iv) *x*−1/2, −*y*+1/2, *z*−1/2; (v) −*x*+1/2, −*y*+1/2, −*z*; (vi) −*x*+1, *y*, −*z*+1/2; (vii) *x*, −*y*, *z*+1/2; (viii) −*x*, *y*, −*z*+1/2; (ix) *x*, −*y*, *z*−1/2.

### trans-Bis(ethylenediamine)(isothiocyanato)nitritocobalt(III) chloride monohydrate (II) Crystal data

**Table:**

[Co(CNS)(NO_2_)(C_2_H_8_N_2_)_2_]Cl·H_2_O	*F*(000) = 696
*M**_r_* = 336.69	*D*_x_ = 1.658 Mg m^−^^3^
Monoclinic, *P*2_1_/*n*	Mo *K*α radiation, λ = 0.71073 Å
*a* = 8.9059 (4) Å	Cell parameters from 9532 reflections
*b* = 12.3302 (5) Å	θ = 2.3–27.9°
*c* = 12.2915 (5) Å	µ = 1.63 mm^−^^1^
β = 92.295 (2)°	*T* = 301 K
*V* = 1348.67 (10) Å^3^	Prism, orange-red
*Z* = 4	0.25 × 0.20 × 0.20 mm

### trans-Bis(ethylenediamine)(isothiocyanato)nitritocobalt(III) chloride monohydrate (II) Data collection

**Table:**

Bruker D8 VENTURE diffractometer	2830 reflections with *I* > 2σ(*I*)
φ and ω scans	*R*_int_ = 0.024
Absorption correction: integration (SADABS; Bruker, 2016)	θ_max_ = 27.9°, θ_min_ = 2.3°
*T*_min_ = 0.697, *T*_max_ = 0.762	*h* = −11→11
14312 measured reflections	*k* = −16→16
3153 independent reflections	*l* = −14→16

### trans-Bis(ethylenediamine)(isothiocyanato)nitritocobalt(III) chloride monohydrate (II) Refinement

**Table:**

Refinement on *F*^2^	Hydrogen site location: mixed
Least-squares matrix: full	H atoms treated by a mixture of independent and constrained refinement
*R*[*F*^2^ > 2σ(*F*^2^)] = 0.032	*w* = 1/[σ^2^(*F*_o_^2^) + (0.0994*P*)^2^ + 0.7693*P*] where *P* = (*F*_o_^2^ + 2*F*_c_^2^)/3
*wR*(*F*^2^) = 0.131	(Δ/σ)_max_ = 0.001
*S* = 0.98	Δρ_max_ = 0.74 e Å^−^^3^
3153 reflections	Δρ_min_ = −0.54 e Å^−^^3^
161 parameters	Extinction correction: SHELXL2014 (Sheldrick 2015b), Fc^*^=kFc[1+0.001xFc^2^λ^3^/sin(2θ)]^-1/4^
3 restraints	Extinction coefficient: 0.018 (3)

### trans-Bis(ethylenediamine)(isothiocyanato)nitritocobalt(III) chloride monohydrate (II) Special details

**Table:**

Geometry. All esds (except the esd in the dihedral angle between two l.s. planes) are estimated using the full covariance matrix. The cell esds are taken into account individually in the estimation of esds in distances, angles and torsion angles; correlations between esds in cell parameters are only used when they are defined by crystal symmetry. An approximate (isotropic) treatment of cell esds is used for estimating esds involving l.s. planes.

### trans-Bis(ethylenediamine)(isothiocyanato)nitritocobalt(III) chloride monohydrate (II) Fractional atomic coordinates and isotropic or equivalent isotropic displacement parameters (Å^2^)

**Table:**

	*x*	*y*	*z*	*U*_iso_*/*U*_eq_	
Co1	0.34572 (3)	0.73903 (2)	0.40502 (2)	0.02724 (16)	
Cl2	0.40898 (8)	1.10470 (6)	0.34687 (5)	0.0491 (2)	
S3	0.38808 (9)	0.91789 (7)	0.07167 (5)	0.0517 (2)	
O4	0.4510 (3)	0.6054 (2)	0.56850 (19)	0.0696 (7)	
O5	0.2569 (3)	0.6935 (3)	0.61042 (18)	0.0682 (7)	
O6	0.6880 (3)	0.9433 (2)	0.29973 (18)	0.0564 (5)	
H6A	0.693 (5)	0.921 (3)	0.237 (2)	0.085*	
H6B	0.615 (4)	0.985 (3)	0.296 (3)	0.085*	
N7	0.3525 (2)	0.67061 (18)	0.54472 (16)	0.0385 (5)	
N8	0.3409 (2)	0.80927 (16)	0.26542 (15)	0.0340 (4)	
N9	0.3774 (2)	0.60117 (16)	0.33151 (17)	0.0352 (4)	
H9A	0.3798	0.5472	0.3796	0.042*	
H9B	0.3030	0.5888	0.2826	0.042*	
N10	0.5649 (3)	0.75194 (18)	0.41100 (19)	0.0371 (5)	
H10A	0.5916	0.8130	0.3784	0.045*	
H10B	0.5985	0.7547	0.4801	0.045*	
N11	0.3149 (2)	0.88049 (17)	0.47145 (17)	0.0377 (4)	
H11A	0.3632	0.8830	0.5362	0.045*	
H11B	0.3525	0.9320	0.4297	0.045*	
N12	0.1264 (2)	0.72780 (19)	0.39439 (19)	0.0370 (5)	
H12A	0.0972	0.6960	0.3321	0.044*	
H12B	0.0937	0.6883	0.4492	0.044*	
C13	0.3590 (2)	0.85381 (19)	0.18492 (18)	0.0320 (5)	
C14	0.5213 (3)	0.6079 (2)	0.2776 (2)	0.0480 (6)	
H14A	0.5104	0.6524	0.2126	0.058*	
H14B	0.5538	0.5361	0.2565	0.058*	
C15	0.6333 (3)	0.6570 (3)	0.3556 (3)	0.0540 (7)	
H15A	0.7206	0.6805	0.3172	0.065*	
H15B	0.6657	0.6035	0.4094	0.065*	
C16	0.1541 (4)	0.9003 (3)	0.4845 (3)	0.0624 (9)	
H16A	0.1330	0.9772	0.4773	0.075*	
H16B	0.1260	0.8773	0.5564	0.075*	
C17	0.0649 (3)	0.8381 (3)	0.3989 (3)	0.0574 (8)	
H17A	−0.0401	0.8356	0.4170	0.069*	
H17B	0.0719	0.8733	0.3287	0.069*	

### trans-Bis(ethylenediamine)(isothiocyanato)nitritocobalt(III) chloride monohydrate (II) Atomic displacement parameters (Å^2^)

**Table:**

	*U*^11^	*U*^22^	*U*^33^	*U*^12^	*U*^13^	*U*^23^
Co1	0.0312 (2)	0.0290 (2)	0.0215 (2)	−0.00349 (10)	−0.00019 (13)	0.00291 (10)
Cl2	0.0641 (4)	0.0417 (4)	0.0398 (4)	−0.0020 (3)	−0.0179 (3)	0.0020 (2)
S3	0.0625 (5)	0.0604 (5)	0.0331 (3)	0.0169 (3)	0.0144 (3)	0.0177 (3)
O4	0.0947 (18)	0.0658 (15)	0.0484 (12)	0.0288 (13)	0.0047 (11)	0.0255 (11)
O5	0.0630 (14)	0.104 (2)	0.0386 (11)	0.0020 (14)	0.0162 (10)	0.0212 (13)
O6	0.0571 (12)	0.0649 (14)	0.0475 (12)	−0.0113 (10)	0.0066 (10)	−0.0063 (10)
N7	0.0471 (11)	0.0404 (11)	0.0281 (9)	−0.0086 (9)	0.0015 (8)	0.0057 (8)
N8	0.0421 (10)	0.0320 (10)	0.0277 (9)	−0.0022 (8)	0.0004 (7)	0.0030 (8)
N9	0.0408 (10)	0.0322 (9)	0.0323 (10)	−0.0002 (8)	−0.0015 (8)	0.0013 (8)
N10	0.0329 (10)	0.0482 (12)	0.0300 (11)	−0.0069 (8)	−0.0014 (8)	0.0054 (8)
N11	0.0471 (11)	0.0352 (10)	0.0305 (10)	−0.0041 (8)	−0.0006 (8)	−0.0019 (8)
N12	0.0331 (10)	0.0462 (12)	0.0319 (11)	−0.0049 (8)	0.0040 (8)	0.0001 (8)
C13	0.0335 (10)	0.0341 (11)	0.0284 (10)	0.0031 (8)	−0.0004 (8)	0.0008 (9)
C14	0.0500 (15)	0.0463 (14)	0.0482 (15)	0.0108 (11)	0.0096 (12)	−0.0018 (12)
C15	0.0379 (13)	0.0566 (17)	0.0676 (19)	0.0027 (12)	0.0031 (12)	0.0009 (15)
C16	0.0541 (17)	0.0601 (19)	0.074 (2)	0.0053 (14)	0.0171 (15)	−0.0243 (17)
C17	0.0408 (14)	0.0608 (18)	0.070 (2)	0.0089 (13)	−0.0001 (13)	−0.0088 (16)

### trans-Bis(ethylenediamine)(isothiocyanato)nitritocobalt(III) chloride monohydrate (II) Geometric parameters (Å, º)

**Table:**

Co1—N7	1.912 (2)	N10—H10B	0.8900
Co1—N8	1.9210 (19)	N11—C16	1.468 (4)
Co1—N11	1.950 (2)	N11—H11A	0.8900
Co1—N9	1.951 (2)	N11—H11B	0.8900
Co1—N10	1.957 (2)	N12—C17	1.468 (4)
Co1—N12	1.957 (2)	N12—H12A	0.8900
S3—C13	1.630 (2)	N12—H12B	0.8900
O4—N7	1.217 (3)	C14—C15	1.485 (4)
O5—N7	1.229 (3)	C14—H14A	0.9700
O6—H6A	0.821 (18)	C14—H14B	0.9700
O6—H6B	0.833 (18)	C15—H15A	0.9700
N8—C13	1.149 (3)	C15—H15B	0.9700
N9—C14	1.468 (3)	C16—C17	1.504 (5)
N9—H9A	0.8900	C16—H16A	0.9700
N9—H9B	0.8900	C16—H16B	0.9700
N10—C15	1.496 (4)	C17—H17A	0.9700
N10—H10A	0.8900	C17—H17B	0.9700
			
N7—Co1—N8	179.20 (8)	C16—N11—H11B	109.6
N7—Co1—N11	91.06 (9)	Co1—N11—H11B	109.6
N8—Co1—N11	88.41 (9)	H11A—N11—H11B	108.1
N7—Co1—N9	91.81 (9)	C17—N12—Co1	107.75 (18)
N8—Co1—N9	88.72 (8)	C17—N12—H12A	110.2
N11—Co1—N9	177.11 (8)	Co1—N12—H12A	110.2
N7—Co1—N10	90.38 (9)	C17—N12—H12B	110.2
N8—Co1—N10	89.06 (9)	Co1—N12—H12B	110.2
N11—Co1—N10	93.92 (9)	H12A—N12—H12B	108.5
N9—Co1—N10	85.73 (9)	N8—C13—S3	178.8 (2)
N7—Co1—N12	91.38 (9)	N9—C14—C15	107.9 (2)
N8—Co1—N12	89.18 (9)	N9—C14—H14A	110.1
N11—Co1—N12	86.24 (9)	C15—C14—H14A	110.1
N9—Co1—N12	94.01 (9)	N9—C14—H14B	110.1
N10—Co1—N12	178.23 (9)	C15—C14—H14B	110.1
H6A—O6—H6B	103 (3)	H14A—C14—H14B	108.4
O4—N7—O5	120.4 (2)	C14—C15—N10	109.7 (2)
O4—N7—Co1	120.22 (18)	C14—C15—H15A	109.7
O5—N7—Co1	119.42 (19)	N10—C15—H15A	109.7
C13—N8—Co1	170.41 (19)	C14—C15—H15B	109.7
C14—N9—Co1	107.74 (16)	N10—C15—H15B	109.7
C14—N9—H9A	110.2	H15A—C15—H15B	108.2
Co1—N9—H9A	110.2	N11—C16—C17	109.2 (2)
C14—N9—H9B	110.2	N11—C16—H16A	109.8
Co1—N9—H9B	110.2	C17—C16—H16A	109.8
H9A—N9—H9B	108.5	N11—C16—H16B	109.8
C15—N10—Co1	110.07 (17)	C17—C16—H16B	109.8
C15—N10—H10A	109.6	H16A—C16—H16B	108.3
Co1—N10—H10A	109.6	N12—C17—C16	108.2 (3)
C15—N10—H10B	109.6	N12—C17—H17A	110.1
Co1—N10—H10B	109.6	C16—C17—H17A	110.1
H10A—N10—H10B	108.2	N12—C17—H17B	110.1
C16—N11—Co1	110.45 (18)	C16—C17—H17B	110.1
C16—N11—H11A	109.6	H17A—C17—H17B	108.4
Co1—N11—H11A	109.6		
			
Co1—N9—C14—C15	44.9 (3)	N9—Co1—N10—C15	2.0 (2)
N9—C14—C15—N10	−44.1 (3)	N12—Co1—N11—C16	3.5 (2)
Co1—N10—C15—C14	22.6 (3)	N11—Co1—N12—C17	21.6 (2)
Co1—N11—C16—C17	−27.5 (3)	O4—N7—Co1—N9	46.3 (2)
Co1—N12—C17—C16	−41.7 (3)	O4—N7—Co1—N10	−39.4 (2)
N11—C16—C17—N12	45.6 (4)	O5—N7—Co1—N11	46.3 (2)
N10—Co1—N9—C14	−26.2 (2)	O5—N7—Co1—N12	−40.0 (2)

### trans-Bis(ethylenediamine)(isothiocyanato)nitritocobalt(III) chloride monohydrate (II) Hydrogen-bond geometry (Å, º)

**Table:**

*D*—H···*A*	*D*—H	H···*A*	*D*···*A*	*D*—H···*A*
O6—H6*A*···O5^i^	0.82 (2)	2.19 (2)	2.958 (3)	156 (4)
O6—H6*B*···Cl2	0.83 (2)	2.45 (2)	3.253 (3)	162 (4)
N9—H9*A*···O4	0.89	2.49	2.960 (3)	114
N9—H9*A*···O4^ii^	0.89	2.48	3.191 (3)	138
N9—H9*B*···Cl2^iii^	0.89	2.43	3.297 (2)	165
N10—H10*A*···O6	0.89	2.08	2.960 (3)	171
N10—H10*B*···Cl2^iv^	0.89	2.75	3.461 (2)	138
N11—H11*A*···Cl2^iv^	0.89	2.44	3.260 (2)	153
N11—H11*B*···Cl2	0.89	2.42	3.285 (2)	164
N12—H12*A*···Cl2^iii^	0.89	2.47	3.335 (2)	164
N12—H12*B*···S3^v^	0.89	2.75	3.585 (2)	157
N12—H12*B*···O5	0.89	2.41	2.887 (3)	114
C15—H15*B*···S3^vi^	0.97	2.77	3.546 (3)	138

Symmetry codes: (i) *x*+1/2, −*y*+3/2, *z*−1/2; (ii) −*x*+1, −*y*+1, −*z*+1; (iii) −*x*+1/2, *y*−1/2, −*z*+1/2; (iv) −*x*+1, −*y*+2, −*z*+1; (v) *x*−1/2, −*y*+3/2, *z*+1/2; (vi) *x*+1/2, −*y*+3/2, *z*+1/2.

### trans-Bis(ethylenediamine)(isothiocyanato)nitritocobalt(III) perchlorate–thiocyanate(0.75/0.25) (III) Crystal data

**Table:**

[Co(CNS)(NO_2_)(C_2_H_8_N_2_)_2_](ClO_4_)_0.75_(CNS)_0.25_	*F*(000) = 1528
*M**_r_* = 372.33	*D*_x_ = 1.759 Mg m^−^^3^*D*_m_ = 1.76 (2) Mg m^−^^3^*D*_m_ measured by flotation
Monoclinic, *P*2_1_/*n*	Mo *K*α radiation, λ = 0.71073 Å
*a* = 11.3141 (6) Å	Cell parameters from 9222 reflections
*b* = 16.2969 (7) Å	θ = 2.5–27.9°
*c* = 16.1298 (7) Å	µ = 1.58 mm^−^^1^
β = 109.023 (2)°	*T* = 301 K
*V* = 2811.7 (2) Å^3^	Prism, orange
*Z* = 8	0.35 × 0.30 × 0.27 mm

### trans-Bis(ethylenediamine)(isothiocyanato)nitritocobalt(III) perchlorate–thiocyanate(0.75/0.25) (III) Data collection

**Table:**

Bruker D8 VENTURE diffractometer	5411 reflections with *I* > 2σ(*I*)
φ and ω scans	*R*_int_ = 0.034
Absorption correction: integration (SADABS; Bruker, 2016)	θ_max_ = 27.9°, θ_min_ = 2.3°
*T*_min_ = 0.544, *T*_max_ = 0.774	*h* = −14→11
30435 measured reflections	*k* = −21→19
6595 independent reflections	*l* = −21→21

### trans-Bis(ethylenediamine)(isothiocyanato)nitritocobalt(III) perchlorate–thiocyanate(0.75/0.25) (III) Refinement

**Table:**

Refinement on *F*^2^	13 restraints
Least-squares matrix: full	Hydrogen site location: inferred from neighbouring sites
*R*[*F*^2^ > 2σ(*F*^2^)] = 0.063	H-atom parameters constrained
*wR*(*F*^2^) = 0.221	*w* = 1/[σ^2^(*F*_o_^2^) + (0.1199*P*)^2^ + 7.1456*P*] where *P* = (*F*_o_^2^ + 2*F*_c_^2^)/3
*S* = 1.10	(Δ/σ)_max_ = 0.001
6595 reflections	Δρ_max_ = 1.40 e Å^−^^3^
379 parameters	Δρ_min_ = −1.15 e Å^−^^3^

### trans-Bis(ethylenediamine)(isothiocyanato)nitritocobalt(III) perchlorate–thiocyanate(0.75/0.25) (III) Special details

**Table:**

Geometry. All esds (except the esd in the dihedral angle between two l.s. planes) are estimated using the full covariance matrix. The cell esds are taken into account individually in the estimation of esds in distances, angles and torsion angles; correlations between esds in cell parameters are only used when they are defined by crystal symmetry. An approximate (isotropic) treatment of cell esds is used for estimating esds involving l.s. planes.

### trans-Bis(ethylenediamine)(isothiocyanato)nitritocobalt(III) perchlorate–thiocyanate(0.75/0.25) (III) Fractional atomic coordinates and isotropic or equivalent isotropic displacement parameters (Å^2^)

**Table:**

	*x*	*y*	*z*	*U*_iso_*/*U*_eq_	Occ. (<1)
Co1	0.26737 (5)	0.24838 (3)	0.26005 (4)	0.02768 (18)	
Co2	0.73662 (5)	0.50800 (3)	0.25489 (4)	0.02803 (18)	
Cl3	1.00831 (11)	0.27237 (9)	0.50358 (8)	0.0488 (3)	
Cl4	0.5000	0.0000	0.5000	0.0959 (11)	
S5	−0.16787 (10)	0.26965 (7)	0.18348 (9)	0.0437 (3)	
S6	1.17122 (10)	0.49090 (7)	0.32114 (9)	0.0424 (3)	
S7	−0.0060 (12)	0.0722 (6)	0.0308 (12)	0.0904 (14)	0.5
O8	0.5136 (3)	0.2946 (3)	0.3191 (3)	0.0638 (11)	
O9	0.4876 (3)	0.1716 (2)	0.2726 (3)	0.0623 (11)	
O10	0.4904 (3)	0.4615 (3)	0.1966 (3)	0.0643 (11)	
O11	0.5155 (3)	0.5834 (2)	0.2431 (3)	0.0677 (12)	
O12	1.0988 (5)	0.3160 (3)	0.4766 (4)	0.0846 (15)	
O13	0.8923 (5)	0.2694 (3)	0.4343 (4)	0.102 (2)	
O14	1.0485 (5)	0.1901 (3)	0.5271 (4)	0.0823 (15)	
O15	0.9910 (6)	0.3161 (4)	0.5742 (4)	0.0996 (19)	
O16	0.5605 (16)	−0.0574 (9)	0.4377 (10)	0.127 (5)	0.5
O17	0.385 (2)	−0.0198 (17)	0.471 (2)	0.225 (15)	0.5
O18	0.5111 (10)	0.0765 (7)	0.4576 (8)	0.083 (3)	0.5
O19	0.588 (3)	−0.0018 (12)	0.5753 (11)	0.165 (10)	0.5
N20	0.4445 (3)	0.2367 (2)	0.2869 (3)	0.0360 (8)	
N21	0.0916 (4)	0.2626 (2)	0.2337 (3)	0.0386 (8)	
N22	0.2391 (4)	0.1979 (2)	0.1448 (3)	0.0406 (8)	
H22A	0.3088	0.1737	0.1431	0.049*	
H22B	0.1793	0.1601	0.1347	0.049*	
N23	0.2773 (3)	0.3522 (2)	0.2017 (2)	0.0349 (7)	
H23A	0.2094	0.3822	0.1964	0.042*	
H23B	0.3439	0.3805	0.2337	0.042*	
N24	0.2970 (4)	0.2984 (2)	0.3751 (2)	0.0405 (8)	
H24A	0.3606	0.3335	0.3865	0.049*	
H24B	0.2294	0.3258	0.3759	0.049*	
N25	0.2551 (3)	0.1441 (2)	0.3166 (3)	0.0392 (8)	
H25A	0.1894	0.1158	0.2835	0.047*	
H25B	0.3236	0.1142	0.3237	0.047*	
N26	0.5597 (3)	0.5190 (2)	0.2292 (3)	0.0358 (8)	
N27	0.9128 (4)	0.4954 (2)	0.2794 (3)	0.0429 (9)	
N28	0.7299 (4)	0.4059 (2)	0.3167 (3)	0.0399 (8)	
H28A	0.6641	0.3764	0.2858	0.048*	
H28B	0.7986	0.3765	0.3229	0.048*	
N29	0.7668 (4)	0.5619 (3)	0.3677 (3)	0.0435 (9)	
H29A	0.8282	0.5984	0.3767	0.052*	
H29B	0.6982	0.5879	0.3688	0.052*	
N30	0.7443 (3)	0.6115 (2)	0.1954 (2)	0.0369 (8)	
H30A	0.6787	0.6426	0.1934	0.044*	
H30B	0.8134	0.6387	0.2249	0.044*	
N31	0.7049 (4)	0.4565 (2)	0.1403 (3)	0.0420 (9)	
H31A	0.7730	0.4303	0.1384	0.050*	
H31B	0.6429	0.4203	0.1305	0.050*	
N32	0.023 (4)	−0.0799 (17)	−0.037 (4)	0.0904 (14)	0.5
C33	−0.0157 (4)	0.2666 (2)	0.2136 (3)	0.0306 (8)	
C34	0.2016 (5)	0.2629 (3)	0.0778 (3)	0.0482 (12)	
H34A	0.1149	0.2781	0.0669	0.058*	
H34B	0.2112	0.2442	0.0233	0.058*	
C35	0.2867 (6)	0.3352 (3)	0.1141 (3)	0.0506 (12)	
H35A	0.3722	0.3220	0.1187	0.061*	
H35B	0.2602	0.3826	0.0761	0.061*	
C36	0.3262 (5)	0.2324 (4)	0.4418 (3)	0.0510 (12)	
H36A	0.3118	0.2513	0.4948	0.061*	
H36B	0.4129	0.2159	0.4566	0.061*	
C37	0.2414 (5)	0.1622 (3)	0.4024 (3)	0.0499 (12)	
H37A	0.2639	0.1145	0.4403	0.060*	
H37B	0.1554	0.1766	0.3952	0.060*	
C38	1.0193 (4)	0.4929 (2)	0.2961 (3)	0.0334 (9)	
C39	0.7204 (6)	0.4248 (4)	0.4024 (4)	0.0604 (15)	
H39A	0.7477	0.3784	0.4416	0.073*	
H39B	0.6345	0.4374	0.3973	0.073*	
C40	0.8022 (7)	0.4973 (4)	0.4369 (4)	0.0625 (16)	
H40A	0.7901	0.5172	0.4902	0.075*	
H40B	0.8894	0.4824	0.4500	0.075*	
C41	0.7442 (7)	0.5947 (4)	0.1067 (4)	0.0650 (16)	
H41A	0.7093	0.6412	0.0692	0.078*	
H41B	0.8293	0.5866	0.1071	0.078*	
C42	0.6706 (7)	0.5219 (4)	0.0729 (4)	0.0652 (16)	
H42A	0.6861	0.5033	0.0203	0.078*	
H42B	0.5823	0.5345	0.0579	0.078*	
C43	−0.0001 (8)	−0.0196 (4)	−0.0072 (6)	0.0321 (18)	0.5

### trans-Bis(ethylenediamine)(isothiocyanato)nitritocobalt(III) perchlorate–thiocyanate(0.75/0.25) (III) Atomic displacement parameters (Å^2^)

**Table:**

	*U*^11^	*U*^22^	*U*^33^	*U*^12^	*U*^13^	*U*^23^
Co1	0.0238 (3)	0.0235 (3)	0.0337 (3)	0.00122 (18)	0.0066 (2)	0.00090 (19)
Co2	0.0250 (3)	0.0225 (3)	0.0343 (3)	−0.00156 (18)	0.0065 (2)	0.00015 (19)
Cl3	0.0382 (6)	0.0488 (7)	0.0563 (7)	−0.0025 (5)	0.0112 (5)	−0.0009 (5)
Cl4	0.0470 (12)	0.091 (2)	0.140 (3)	0.0020 (12)	0.0172 (15)	0.0573 (19)
S5	0.0256 (5)	0.0382 (6)	0.0623 (7)	0.0035 (4)	0.0076 (5)	0.0011 (5)
S6	0.0286 (5)	0.0395 (6)	0.0570 (7)	0.0038 (4)	0.0108 (5)	0.0017 (5)
S7	0.066 (4)	0.098 (3)	0.105 (3)	−0.008 (3)	0.024 (3)	0.005 (2)
O8	0.0335 (18)	0.059 (2)	0.094 (3)	−0.0142 (17)	0.0139 (19)	−0.015 (2)
O9	0.0354 (18)	0.054 (2)	0.094 (3)	0.0127 (16)	0.0170 (19)	−0.015 (2)
O10	0.0347 (19)	0.056 (2)	0.099 (3)	−0.0186 (17)	0.0176 (19)	−0.015 (2)
O11	0.0360 (19)	0.045 (2)	0.118 (4)	0.0091 (16)	0.020 (2)	−0.011 (2)
O12	0.089 (4)	0.074 (3)	0.112 (4)	−0.021 (3)	0.062 (3)	−0.005 (3)
O13	0.064 (3)	0.078 (3)	0.122 (5)	−0.003 (3)	−0.026 (3)	0.008 (3)
O14	0.070 (3)	0.060 (3)	0.114 (4)	0.016 (2)	0.025 (3)	0.030 (3)
O15	0.114 (5)	0.106 (4)	0.108 (4)	−0.024 (4)	0.076 (4)	−0.035 (3)
O16	0.174 (15)	0.093 (9)	0.122 (11)	0.051 (10)	0.059 (10)	0.014 (8)
O17	0.127 (13)	0.24 (3)	0.28 (3)	−0.112 (17)	0.029 (18)	0.07 (2)
O18	0.075 (6)	0.070 (6)	0.121 (9)	0.015 (5)	0.053 (6)	0.030 (6)
O19	0.26 (3)	0.128 (15)	0.086 (10)	0.104 (17)	0.023 (13)	0.018 (9)
N20	0.0240 (17)	0.0368 (18)	0.044 (2)	−0.0002 (14)	0.0068 (15)	0.0006 (15)
N21	0.0287 (18)	0.0368 (19)	0.046 (2)	0.0032 (14)	0.0060 (15)	0.0037 (15)
N22	0.0374 (19)	0.0339 (19)	0.046 (2)	0.0003 (15)	0.0068 (16)	−0.0047 (15)
N23	0.0337 (18)	0.0302 (17)	0.0416 (19)	−0.0003 (14)	0.0135 (15)	−0.0001 (14)
N24	0.0354 (19)	0.041 (2)	0.0406 (19)	−0.0017 (16)	0.0066 (15)	−0.0010 (16)
N25	0.0322 (18)	0.0327 (18)	0.053 (2)	0.0022 (14)	0.0151 (16)	0.0091 (16)
N26	0.0259 (17)	0.0320 (17)	0.046 (2)	−0.0028 (13)	0.0064 (14)	−0.0003 (15)
N27	0.033 (2)	0.038 (2)	0.054 (2)	0.0023 (15)	0.0097 (17)	0.0029 (17)
N28	0.0370 (19)	0.0314 (18)	0.052 (2)	0.0033 (15)	0.0159 (16)	0.0092 (15)
N29	0.039 (2)	0.043 (2)	0.043 (2)	−0.0007 (16)	0.0052 (16)	−0.0066 (16)
N30	0.0345 (18)	0.0294 (17)	0.048 (2)	0.0007 (14)	0.0142 (15)	0.0024 (15)
N31	0.038 (2)	0.0343 (19)	0.049 (2)	0.0010 (15)	0.0083 (16)	−0.0065 (16)
N32	0.066 (4)	0.098 (3)	0.105 (3)	−0.008 (3)	0.024 (3)	0.005 (2)
C33	0.032 (2)	0.0264 (18)	0.0313 (19)	−0.0003 (15)	0.0079 (16)	−0.0003 (14)
C34	0.052 (3)	0.054 (3)	0.035 (2)	0.006 (2)	0.009 (2)	−0.001 (2)
C35	0.064 (3)	0.046 (3)	0.047 (3)	−0.004 (2)	0.025 (2)	0.008 (2)
C36	0.047 (3)	0.065 (3)	0.035 (2)	0.006 (2)	0.006 (2)	0.004 (2)
C37	0.050 (3)	0.050 (3)	0.054 (3)	0.004 (2)	0.024 (2)	0.022 (2)
C38	0.032 (2)	0.0285 (19)	0.037 (2)	0.0003 (15)	0.0071 (16)	−0.0009 (15)
C39	0.078 (4)	0.052 (3)	0.056 (3)	0.003 (3)	0.027 (3)	0.016 (2)
C40	0.067 (4)	0.078 (4)	0.035 (3)	−0.005 (3)	0.006 (2)	0.006 (2)
C41	0.096 (5)	0.050 (3)	0.055 (3)	0.006 (3)	0.033 (3)	0.013 (2)
C42	0.079 (4)	0.071 (4)	0.043 (3)	−0.013 (3)	0.016 (3)	−0.002 (3)
C43	0.027 (3)	0.025 (3)	0.045 (3)	−0.004 (3)	0.012 (2)	−0.003 (3)

### trans-Bis(ethylenediamine)(isothiocyanato)nitritocobalt(III) perchlorate–thiocyanate(0.75/0.25) (III) Geometric parameters (Å, º)

**Table:**

Co1—N21	1.907 (4)	N25—C37	1.471 (6)
Co1—N20	1.916 (4)	N25—H25A	0.8900
Co1—N24	1.953 (4)	N25—H25B	0.8900
Co1—N25	1.956 (4)	N27—C38	1.147 (6)
Co1—N23	1.957 (3)	N28—C39	1.455 (7)
Co1—N22	1.961 (4)	N28—H28A	0.8900
Co2—N27	1.912 (4)	N28—H28B	0.8900
Co2—N26	1.915 (4)	N29—C40	1.491 (7)
Co2—N29	1.947 (4)	N29—H29A	0.8900
Co2—N31	1.954 (4)	N29—H29B	0.8900
Co2—N28	1.954 (4)	N30—C41	1.455 (7)
Co2—N30	1.957 (4)	N30—H30A	0.8900
Cl3—O15	1.410 (5)	N30—H30B	0.8900
Cl3—O13	1.421 (5)	N31—C42	1.480 (7)
Cl3—O12	1.426 (5)	N31—H31A	0.8900
Cl3—O14	1.427 (4)	N31—H31B	0.8900
Cl4—O17	1.27 (2)	N32—C43	1.158 (4)
Cl4—O19	1.30 (2)	C34—C35	1.514 (8)
Cl4—O18	1.447 (10)	C34—H34A	0.9700
Cl4—O16	1.674 (13)	C34—H34B	0.9700
S5—C33	1.630 (4)	C35—H35A	0.9700
S6—C38	1.633 (4)	C35—H35B	0.9700
S7—C43	1.627 (4)	C36—C37	1.496 (8)
O8—N20	1.227 (5)	C36—H36A	0.9700
O9—N20	1.220 (5)	C36—H36B	0.9700
O10—N26	1.224 (5)	C37—H37A	0.9700
O11—N26	1.215 (5)	C37—H37B	0.9700
N21—C33	1.152 (6)	C39—C40	1.491 (9)
N22—C34	1.473 (6)	C39—H39A	0.9700
N22—H22A	0.8900	C39—H39B	0.9700
N22—H22B	0.8900	C40—H40A	0.9700
N23—C35	1.479 (6)	C40—H40B	0.9700
N23—H23A	0.8900	C41—C42	1.451 (9)
N23—H23B	0.8900	C41—H41A	0.9700
N24—C36	1.481 (6)	C41—H41B	0.9700
N24—H24A	0.8900	C42—H42A	0.9700
N24—H24B	0.8900	C42—H42B	0.9700
			
N21—Co1—N20	178.72 (16)	C39—N28—H28A	109.8
N21—Co1—N24	90.29 (17)	Co2—N28—H28A	109.8
N20—Co1—N24	89.06 (16)	C39—N28—H28B	109.8
N21—Co1—N25	88.97 (16)	Co2—N28—H28B	109.8
N20—Co1—N25	92.09 (16)	H28A—N28—H28B	108.3
N24—Co1—N25	86.33 (17)	C40—N29—Co2	107.7 (3)
N21—Co1—N23	90.39 (16)	C40—N29—H29A	110.2
N20—Co1—N23	88.56 (16)	Co2—N29—H29A	110.2
N24—Co1—N23	94.38 (16)	C40—N29—H29B	110.2
N25—Co1—N23	179.05 (16)	Co2—N29—H29B	110.2
N21—Co1—N22	90.19 (17)	H29A—N29—H29B	108.5
N20—Co1—N22	90.46 (17)	C41—N30—Co2	109.5 (3)
N24—Co1—N22	179.51 (16)	C41—N30—H30A	109.8
N25—Co1—N22	93.62 (17)	Co2—N30—H30A	109.8
N23—Co1—N22	85.67 (16)	C41—N30—H30B	109.8
N27—Co2—N26	179.05 (17)	Co2—N30—H30B	109.8
N27—Co2—N29	90.07 (18)	H30A—N30—H30B	108.2
N26—Co2—N29	90.74 (17)	C42—N31—Co2	108.0 (3)
N27—Co2—N31	90.42 (18)	C42—N31—H31A	110.1
N26—Co2—N31	88.77 (17)	Co2—N31—H31A	110.1
N29—Co2—N31	178.61 (17)	C42—N31—H31B	110.1
N27—Co2—N28	90.79 (17)	Co2—N31—H31B	110.1
N26—Co2—N28	88.78 (16)	H31A—N31—H31B	108.4
N29—Co2—N28	86.09 (17)	N21—C33—S5	178.3 (4)
N31—Co2—N28	95.20 (17)	N22—C34—C35	106.4 (4)
N27—Co2—N30	89.11 (16)	N22—C34—H34A	110.5
N26—Co2—N30	91.34 (16)	C35—C34—H34A	110.5
N29—Co2—N30	92.72 (17)	N22—C34—H34B	110.5
N31—Co2—N30	85.99 (16)	C35—C34—H34B	110.5
N28—Co2—N30	178.80 (16)	H34A—C34—H34B	108.7
O15—Cl3—O13	108.5 (4)	N23—C35—C34	106.3 (4)
O15—Cl3—O12	107.3 (3)	N23—C35—H35A	110.5
O13—Cl3—O12	110.4 (4)	C34—C35—H35A	110.5
O15—Cl3—O14	112.1 (4)	N23—C35—H35B	110.5
O13—Cl3—O14	108.0 (3)	C34—C35—H35B	110.5
O12—Cl3—O14	110.6 (3)	H35A—C35—H35B	108.7
O17—Cl4—O19	136.1 (14)	N24—C36—C37	106.6 (4)
O17—Cl4—O18	106.4 (12)	N24—C36—H36A	110.4
O19—Cl4—O18	107.6 (11)	C37—C36—H36A	110.4
O17—Cl4—O16	102.0 (16)	N24—C36—H36B	110.4
O19—Cl4—O16	102.0 (10)	C37—C36—H36B	110.4
O18—Cl4—O16	94.7 (6)	H36A—C36—H36B	108.6
O9—N20—O8	120.6 (4)	N25—C37—C36	107.7 (4)
O9—N20—Co1	119.7 (3)	N25—C37—H37A	110.2
O8—N20—Co1	119.7 (3)	C36—C37—H37A	110.2
C33—N21—Co1	175.1 (4)	N25—C37—H37B	110.2
C34—N22—Co1	108.2 (3)	C36—C37—H37B	110.2
C34—N22—H22A	110.1	H37A—C37—H37B	108.5
Co1—N22—H22A	110.1	N27—C38—S6	178.8 (5)
C34—N22—H22B	110.1	N28—C39—C40	107.1 (5)
Co1—N22—H22B	110.1	N28—C39—H39A	110.3
H22A—N22—H22B	108.4	C40—C39—H39A	110.3
C35—N23—Co1	109.3 (3)	N28—C39—H39B	110.3
C35—N23—H23A	109.8	C40—C39—H39B	110.3
Co1—N23—H23A	109.8	H39A—C39—H39B	108.6
C35—N23—H23B	109.8	N29—C40—C39	107.3 (4)
Co1—N23—H23B	109.8	N29—C40—H40A	110.3
H23A—N23—H23B	108.3	C39—C40—H40A	110.3
C36—N24—Co1	108.4 (3)	N29—C40—H40B	110.3
C36—N24—H24A	110.0	C39—C40—H40B	110.3
Co1—N24—H24A	110.0	H40A—C40—H40B	108.5
C36—N24—H24B	110.0	C42—C41—N30	109.9 (5)
Co1—N24—H24B	110.0	C42—C41—H41A	109.7
H24A—N24—H24B	108.4	N30—C41—H41A	109.7
C37—N25—Co1	108.0 (3)	C42—C41—H41B	109.7
C37—N25—H25A	110.1	N30—C41—H41B	109.7
Co1—N25—H25A	110.1	H41A—C41—H41B	108.2
C37—N25—H25B	110.1	C41—C42—N31	109.4 (5)
Co1—N25—H25B	110.1	C41—C42—H42A	109.8
H25A—N25—H25B	108.4	N31—C42—H42A	109.8
O11—N26—O10	119.7 (4)	C41—C42—H42B	109.8
O11—N26—Co2	120.3 (3)	N31—C42—H42B	109.8
O10—N26—Co2	119.9 (3)	H42A—C42—H42B	108.2
C38—N27—Co2	175.6 (4)	N32—C43—S7	168 (2)
C39—N28—Co2	109.3 (3)		
			
Co1—N22—C34—C35	42.9 (5)	N25—Co1—N24—C36	14.3 (3)
Co1—N23—C35—C34	38.5 (5)	N24—Co1—N25—C37	14.4 (3)
N22—C34—C35—N23	−53.2 (5)	N29—Co2—N28—C39	13.3 (4)
Co1—N24—C36—C37	−39.4 (5)	N28—Co2—N29—C40	15.3 (4)
Co1—N25—C37—C36	−40.0 (5)	N31—Co2—N30—C41	−9.3 (4)
N24—C36—C37—N25	52.3 (5)	N30—Co2—N31—C42	−15.1 (4)
Co2—N28—C39—C40	−38.7 (5)	O8—N20—Co1—N23	48.4 (4)
Co2—N29—C40—C39	−40.3 (6)	O8—N20—Co1—N24	−46.0 (4)
N28—C39—C40—N29	51.9 (7)	O9—N20—Co1—N22	−46.7 (4)
Co2—N30—C41—C42	32.7 (6)	O9—N20—Co1—N25	47.0 (4)
N30—C41—C42—N31	−46.2 (7)	O10—N26—Co2—N28	−50.7 (4)
Co2—N31—C42—C41	37.1 (6)	O10—N26—Co2—N31	44.5 (4)
N23—Co1—N22—C34	−17.6 (3)	O11—N26—Co2—N29	44.9 (4)
N22—Co1—N23—C35	−12.2 (3)	O11—N26—Co2—N30	−47.9 (4)

### trans-Bis(ethylenediamine)(isothiocyanato)nitritocobalt(III) perchlorate–thiocyanate(0.75/0.25) (III) Hydrogen-bond geometry (Å, º)

**Table:**

*D*—H···*A*	*D*—H	H···*A*	*D*···*A*	*D*—H···*A*
N22—H22*A*···O9	0.89	2.39	2.925 (5)	119
N22—H22*B*···S7	0.89	2.64	3.456 (12)	153
N23—H23*A*···S6^i^	0.89	2.82	3.429 (4)	127
N23—H23*B*···O8	0.89	2.41	2.884 (5)	113
N23—H23*B*···O10	0.89	2.34	3.021 (5)	133
N24—H24*A*···S7^ii^	0.89	2.79	3.475 (18)	135
N24—H24*A*···O8	0.89	2.41	2.875 (6)	113
N24—H24*B*···S6^i^	0.89	2.84	3.437 (4)	126
N24—H24*B*···O12^i^	0.89	2.53	3.192 (6)	132
N25—H25*A*···O11^iii^	0.89	2.28	3.059 (5)	147
N25—H25*B*···O9	0.89	2.45	2.973 (5)	118
N25—H25*B*···O18	0.89	2.56	3.237 (12)	133
N25—H25*B*···O19^iv^	0.89	2.44	3.089 (15)	130
N28—H28*A*···O8	0.89	2.36	3.057 (5)	135
N28—H28*A*···O10	0.89	2.45	2.915 (6)	113
N28—H28*B*···O13	0.89	2.48	3.107 (6)	128
N29—H29*A*···O15^v^	0.89	2.39	3.266 (7)	170
N29—H29*B*···O11	0.89	2.38	2.919 (6)	119
N30—H30*A*···O11	0.89	2.44	2.967 (6)	118
N30—H30*B*···O9^vi^	0.89	2.30	3.067 (5)	144
N31—H31*A*···S5^vii^	0.89	2.74	3.343 (4)	126
N31—H31*A*···O16^vi^	0.89	2.57	3.294 (17)	139
N31—H31*B*···O10	0.89	2.40	2.856 (6)	112
N31—H31*B*···O14^viii^	0.89	2.45	3.172 (6)	139
C35—H35*B*···O17^ix^	0.97	2.24	3.09 (2)	145
C36—H36*A*···O12^i^	0.97	2.56	3.119 (8)	117
C36—H36*B*···O18	0.97	2.53	3.249 (11)	131
C37—H37*A*···O16^iv^	0.97	2.48	3.290 (15)	141
C37—H37*A*···O19^iv^	0.97	2.55	3.197 (17)	124
C39—H39*A*···O13	0.97	2.44	3.133 (8)	128
C41—H41*A*···O13^vi^	0.97	2.38	3.202 (8)	143
C41—H41*B*···O18^vi^	0.97	2.37	3.272 (12)	155

Symmetry codes: (i) *x*−1, *y*, *z*; (ii) *x*+1/2, −*y*+1/2, *z*+1/2; (iii) −*x*+1/2, *y*−1/2, −*z*+1/2; (iv) −*x*+1, −*y*, −*z*+1; (v) −*x*+2, −*y*+1, −*z*+1; (vi) −*x*+3/2, *y*+1/2, −*z*+1/2; (vii) *x*+1, *y*, *z*; (viii) *x*−1/2, −*y*+1/2, *z*−1/2; (ix) −*x*+1/2, *y*+1/2, −*z*+1/2.
